# Supplementary material for: NO2, BC and PM Exposure of Participants in the Polluscope Autumn 2019 Campaign in the Paris Region
Source: Toxics. 2023 Feb 23;11(3):206. doi: 10.3390/toxics11030206 (PMC10051186; doi:10.3390/toxics11030206)
Supplement: Supplementary file 1 [file toxics-11-00206-s001.zip › toxics-2142254-supplementary.pdf]

Table S1. Table of results of the Outilset algorithm for Canarin for PM1. * sensor used for complementary experiments.

Instruments	Mean Value	Match	RMSE	Pearson	Kendall	Spearman	Presence	LFE	IPI
CANARIN__01	9.85	0.62	3.77	0.94	0.86	0.96	0.63	1.00	0.80
CANARIN__02	9.47	0.73	3.41	0.94	0.84	0.95	0.99	1.00	0.87
CANARIN__03	11.04	0.64	4.20	0.92	0.81	0.94	0.78	1.00	0.82
CANARIN__04	10.12	0.67	4.03	0.93	0.82	0.94	1.00	1.00	0.85
CANARIN__05	9.03	0.50	2.58	0.93	0.85	0.96	0.92	1.00	0.84
CANARIN__06	9.45	0.62	3.25	0.94	0.83	0.95	1.00	1.00	0.86
CANARIN__10*	11.57	0.58	4.66	0.94	0.83	0.95	0.55	1.00	0.78
CANARIN__12	9.95	0.50	3.82	0.93	0.84	0.96	0.64	1.00	0.78
CANARIN__16	10.18	0.64	3.93	0.94	0.84	0.95	1.00	1.00	0.85
CANARIN__17	10.10	0.76	3.93	0.90	0.81	0.93	1.00	1.00	0.86
Mean	10.08	0.63	3.76	0.93	0.83	0.95	0.85	1.00	0.83

Table S2. Table of results of the Outilset algorithm for Canarin for PM2.5. * sensor used for complementary experiments.

Instruments	Mean Value	Match	RMSE	Pearson	Kendall	Spearman	Presence	LFE	IPI
CANARIN__01	13.71	0.77	7.14	0.94	0.84	0.96	0.63	1.00	0.80
CANARIN__02	13.15	0.75	6.72	0.94	0.83	0.95	0.99	1.00	0.85
CANARIN__03	16.65	0.70	8.90	0.92	0.79	0.93	0.78	1.00	0.80
CANARIN__04	13.87	0.74	6.86	0.93	0.81	0.94	1.00	1.00	0.85
CANARIN__05	12.60	0.76	5.50	0.93	0.84	0.96	0.92	1.00	0.85
CANARIN__06	13.75	0.76	6.86	0.94	0.83	0.95	1.00	1.00	0.85
CANARIN__10*	17.38	0.71	9.80	0.94	0.82	0.95	0.55	1.00	0.77
CANARIN__12	15.31	0.75	8.57	0.93	0.83	0.95	0.63	1.00	0.79
CANARIN__16	15.29	0.74	8.39	0.93	0.82	0.94	1.00	1.00	0.84
CANARIN__17	13.56	0.73	6.72	0.91	0.80	0.93	1.00	1.00	0.84
Mean	14.53	0.74	7.55	0.93	0.82	0.95	0.85	1.00	0.82

Table S3. Table of results of the Outilset algorithm for Canarin for PM10. * sensor used for complementary experiments.

Instruments	Mean Value	Match	RMSE	Pearson	Kendall	Spearman	Presence	LFE	IPI
CANARIN__01	14.44	0.56	6.51	0.84	0.65	0.83	0.63	1.00	0.72
CANARIN__02	14.12	0.54	6.52	0.85	0.64	0.81	1.00	1.00	0.77
CANARIN__03	18.01	0.56	8.34	0.80	0.55	0.73	0.78	1.00	0.71
CANARIN__04	15.21	0.58	6.61	0.83	0.63	0.81	1.00	1.00	0.77
CANARIN__05	13.52	0.63	5.20	0.86	0.69	0.87	0.92	1.00	0.80
CANARIN__06	15.70	0.54	7.62	0.83	0.62	0.80	1.00	1.00	0.76
CANARIN__10*	19.24	0.50	10.07	0.82	0.65	0.84	0.55	1.00	0.69
CANARIN__12	16.69	0.60	8.25	0.83	0.63	0.81	0.63	1.00	0.72
CANARIN__16	16.60	0.58	7.86	0.84	0.63	0.81	1.00	1.00	0.77
CANARIN__17	14.82	0.57	6.49	0.81	0.62	0.79	1.00	1.00	0.76

Mean	15.84	0.57	7.35	0.83	0.63	0.81	0.85	1.00	0.75

Table S4. Table of results of the Outilset algorithm for AE51. * sensor used for complementary experiments.

Instruments	Mean Value	Match	RMSE	Pearson	Kendall	Spearman	Presence	LFE	IPI
AE51_S0_114	578.81	0.71	84.45	0.97	0.78	0.93	1.00	0.99	0.89
AE51_S6_1241	666.54	0.77	104.85	0.98	0.85	0.96	1.00	0.99	0.91
AE51_S6_1242	662.81	0.63	118.62	0.96	0.77	0.92	1.00	0.99	0.87
AE51_S6_1244	672.05	0.61	169.56	0.99	0.82	0.94	0.73	1.00	0.83
AE51_S6_1247*	699.94	0.74	140.15	0.98	0.81	0.94	1.00	0.99	0.90
AE51_S6_1248	705.94	0.77	139.90	0.98	0.84	0.96	1.00	0.99	0.91
Mean	664.35	0.70	126.26	0.98	0.81	0.94	0.95	0.99	0.89

Table S5. Table of results of the Outilset algorithm for Cairsens. * sensor used for complementary experiments.

Instruments	Mean Value	Match	RMSE	Pearson	Kendall	Spearman	Presence	LFE	IPI
Data_3749	33.1	0.54	11.92	0.67	0.50	0.69	0.98	0.99	0.72
Data_3750 *	37.5	0.57	13.35	0.68	0.51	0.70	1.00	1.00	0.73
Data_3754	34.4	0.54	11.76	0.68	0.51	0.70	1.00	1.00	0.73
Data_3755	33.2	0.27	11.16	0.69	0.53	0.73	0.73	1.00	0.66
Data_3756	40.9	0.60	15.78	0.69	0.52	0.71	1.00	1.00	0.73
Data_3970	27.1	0.47	12.07	0.66	0.49	0.68	1.00	0.99	0.69
Data_3971	28.1	0.61	10.07	0.68	0.51	0.69	1.00	1.00	0.73
Data_3972	22.5	0.57	12.80	0.67	0.50	0.68	1.00	0.99	0.69
Data_3974	29.7	0.49	12.19	0.65	0.49	0.67	1.00	0.99	0.70
Data_3975	27.7	0.45	12.32	0.65	0.49	0.67	1.00	0.99	0.69
Data_3977	22.7	0.54	12.33	0.66	0.49	0.67	1.00	1.00	0.69
Data_3978	25.0	0.26	14.11	0.63	0.58	0.77	0.56	0.99	0.60
Data_3979	33.2	0.54	12.31	0.66	0.49	0.68	1.00	0.99	0.71
Data_3982	33.3	0.33	14.93	0.63	0.52	0.71	0.73	0.99	0.64
Data_3983	21.0	0.54	13.70	0.65	0.48	0.67	1.00	0.99	0.67
Mean	30.0	0.5	12.7	0.7	0.5	0.7	0.9	1.0	0.7

Table S6. Limit values of pollutants chosen for the algorithm

Pollutants	Limit values
NO_2	250 ppb
PM	300 µg.m^{-3}
BC	50,000 ng.m^{-3}<br>−1500 ng.m^{-3}

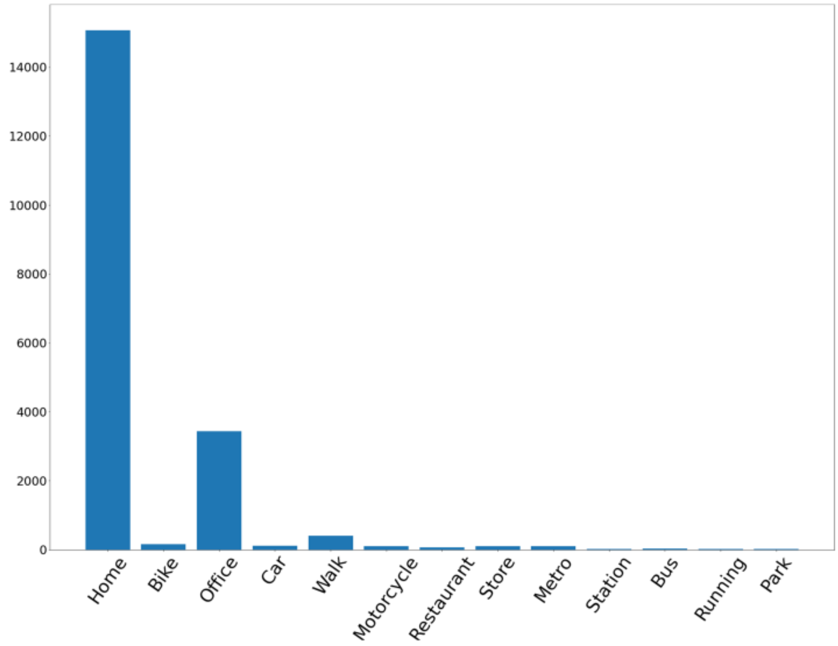

Figure S1 : Distribution of data over classes before class balancing

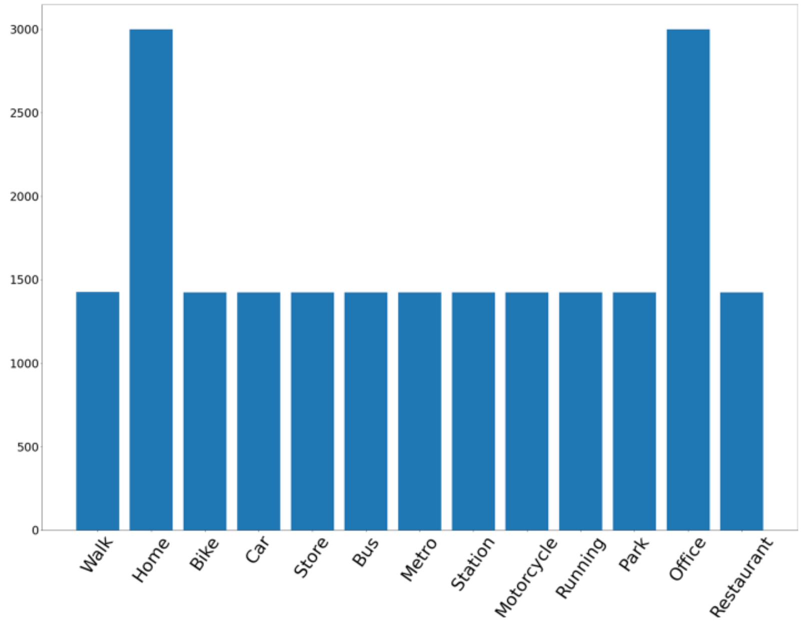

Figure S1 : Distribution of data over classes after class balancing

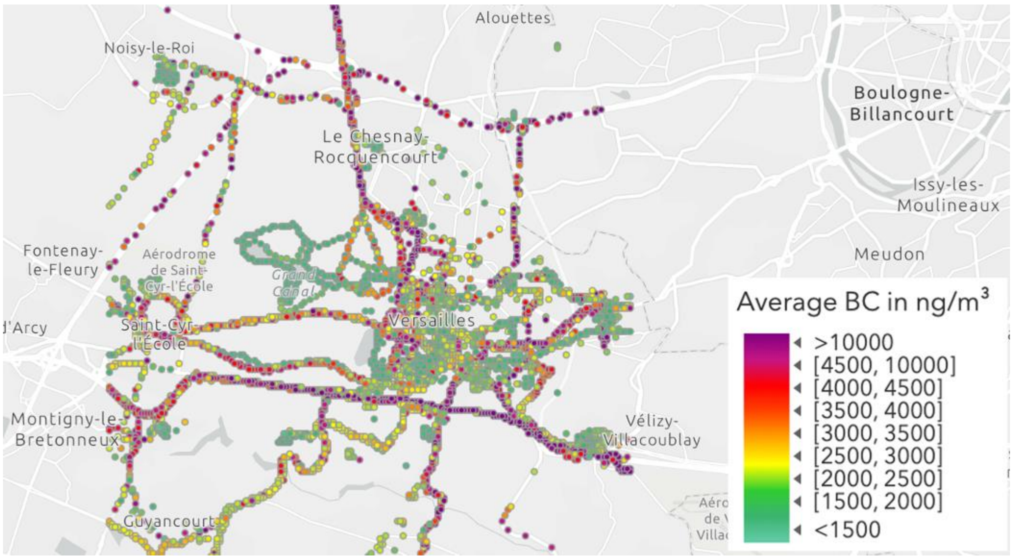

Figure S3. Geolocated BC concentration in Versailles

Table S7. Summary of concentrations for participant 71

		**PM1.0** (µg.m-3)	**PM2.5** (µg.m-3)	**PM10** (µg.m-3)	**NO2** (µg.m-3)	**BC** (ng.m-3)
Day	Mean	11.7	17.9	19.8	13.8	968.7
Hour	Mean (STD)	12.2 (9.9)	18.8 (15)	20.7 (16.4)	14.3 (17)	922.3 (1434)
	Median	10	15	16	7.6	459
Minute	p95	40.0	59.0	64.0	19.0	4487.9

S1. Experiments in specific environments

The experiments presented here are those conducted in indoor, in subway and in car.

S1.1 Inside air

Figure S2 shows the results obtained during the indoor experiment with the different phases of cooking, burning incense and window opening in order to determine their effect on air pollutant concentration. During the cooking activity, electric hotplates were used and no additional ventilation system was activated.

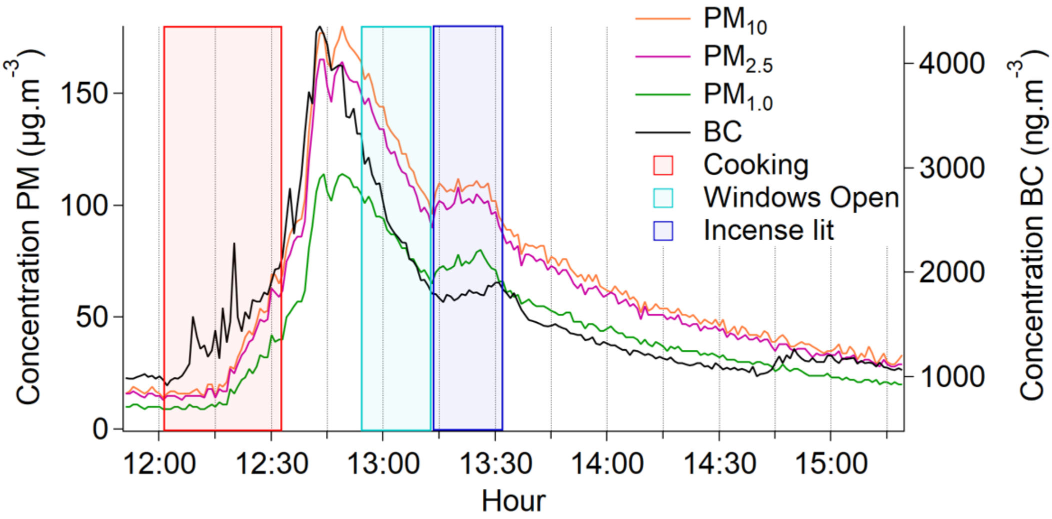

Figure S4. Concentration measured during the indoor for BC and PM.

PM and BC concentrations increased drastically during cooking up to more than 100 µg.m^{-3} for all PM and more than 4000 ng.m^{-3} for BC (Table S8). They continued to increase even after cooking and only started to decrease when the windows were opened. There is therefore a coincidence between cooking and the increase in concentration [1]. When incense was burnt, PM and BC increased, but less than during cooking, showing also a coincidence between incense burning and increased concentration [2]. This shows that specific indoor activities, such as cooking or candle burning, can lead to very high exposition to BC and PM, which can be lowered by ventilation.

S1.2. Subway

Figure S3 shows the results obtained during the subway experiment.

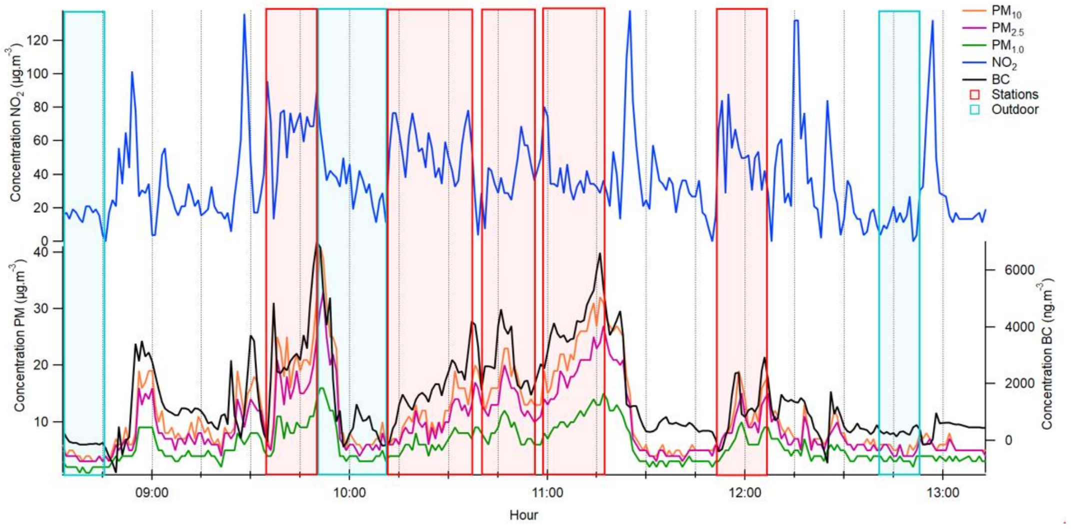

Figure S5. Concentration measured during the subway experiment. NO_2 is on the upper panel, PM and BC are on the bottom panel

Table S9. Mean of concentrations measured during the subway experiment

Pollutants	Mean Outdoor	Mean Stations	Mean Transport
PM_1 ($\mu g.m^{-3}$)	4	8	5
$PM_{2.5}$ ($\mu g.m^{-3}$)	6	14	8
PM_{10} ($\mu g.m^{-3}$)	7	17	10
PM_1/PM_{10}	0.55	0.49	0.53
NO_2 ($\mu g.m^{-3}$)	27	45	50
BC ($ng.m^{-3}$)	480	2561	1318

Figure S9 shows the measured concentrations for the different pollutants. The red and blue boxes refer respectively to periods of outdoor air and inside metro stations.

As shown in Figure S9 and Table 4, outdoor concentrations are the lowest for all pollutants, while in transport, concentrations increased for all pollutants. The highest average concentrations were for NO_2, with value up to 50 $\mu g.m^{-3}$ (Table S9). There are only a few sources of NO2 in the metro (like the engines of thermal machines which sometimes work at night), the high levels are likely linked to the location of air intakes which may be close to road traffic. The concentrations of PM and BC were highest in railway stations. This may due to the piston effect that resuspends the compounds when a train passes and to the accumulation of pollutants [3].

S1.3. Car

Figures S4 shows the results obtained with the car experiment. The experiment was carried out in a diesel car during the week. Highways and city roads were used in this experiment.

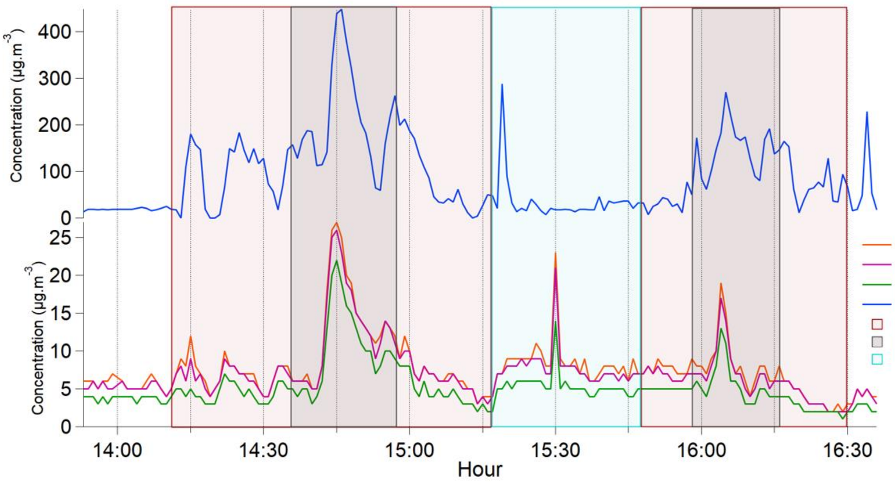

Figure S6. Concentration measured during the car experiment. NO_2 is on the upper panel, PM are on the bottom panel

Table S10. Mean of concentrations measured during the car experiment

Pollutants	Background concentration	Engine on	Engine off	Traffic jam
PM_1 ($\mu g.m^{-3}$)	4	6	5	9
$PM_{2.5}$ ($\mu g.m^{-3}$)	6	8	8	11
PM_{10} ($\mu g.m^{-3}$)	6	8	8	12
PM_1/PM_{10}	0.66	0.70	0.64	0.73

NO_2 ($\mu g.m^{-3}$)	18	121	34	184
BC ($\mu g.m^{-3}$)	1	6	1	11

The concentrations of the different pollutants increased especially during traffic jams (figure S4 and Table S10). NO_2 concentrations are much higher than PM and BC even when the engine was turned off. PM concentrations presented the least variation during traffic jams and when the engine was off (Table S10). NO_2 concentrations were much higher when the engine was turned on, these high concentrations are mostly related to the emissions of the vehicles in front.

1. Lee, J.-B., Kim, K.-H., Kim, H.-J., Cho, S.-J., Jung, K., Kim, S.-D.: Emission Rate of Particulate Matter and Its Removal Efficiency by Precipitators in Under-Fired Charbroiling Restaurants. The Scientific World JOURNAL. 11, 1077–1088 (2011). https://doi.org/10.1100/tsw.2011.103
2. Cattaneo, A., Tecce, N., Derudi, M., Gelosa, S., Nano, G., Cavallo, D.M.: Assessment of Modeled Indoor Air Concentrations of Particulate Matter, Gaseous Pollutants, and Volatile Organic Compounds Emitted from Candles. Human and Ecological Risk Assessment: An International Journal. 20, 962–979 (2014). https://doi.org/10.1080/10807039.2013.821902
3. Cha, Y., Tu, M., Elmgren, M., Silvergren, S., Olofsson, U.: Variation in Airborne Particulate Levels at a Newly Opened Underground Railway Station. Aerosol Air Qual. Res. 19, 737–748 (2019). https://doi.org/10.4209/aaqr.2018.06.0225
